# Projection scenarios of body mass index (2013–2030) for Public Health Planning in Quebec

**DOI:** 10.1186/1471-2458-14-996

**Published:** 2014-09-25

**Authors:** Ernest Lo, Denis Hamel, Yun Jen, Patricia Lamontagne, Sylvie Martel, Colin Steensma, Chantal Blouin, Russell Steele

**Affiliations:** Institut National de Santé Publique du Québec, 190 blvd Crémazie Est, Montréal, Québec H2P 1E2 Canada; Department of Epidemiology, Biostatistics and Occupational Health, McGill University, Purvis Hall, 1020 Pine Avenue West, Montréal, Québec H3A 1A2 Canada; Public Health Agency of Canada, 200, boulevard René-Lévesque Ouest, Montréal, Québec H2Z 1X4 Canada; Department of Mathematics and Statistics, McGill University, 805 Sherbrooke Ouest, Montreal, Québec H3A 2K6 Canada

**Keywords:** Projections, Forecasting, Obesity, Body mass index, Type 2 diabetes, Health targets, Public health, Public health planning, Health burden

## Abstract

**Background:**

Projection analyses can provide estimates of the future health burden of increasing BMI and represent a relevant and useful tool for public health planning. Our study presents long-term (2013–2030) projections of the prevalence and numbers of individuals by BMI category for adult men and women in Quebec. Three applications of projections to estimate outcomes more directly pertinent to public health planning, as well as an in-depth discussion of limits, are provided with the aim of encouraging greater use of projection analyses by public health officers.

**Methods:**

The weighted compositional regression method is applied to prevalence time series derived from sixteen cross-sectional survey cycles, for scenarios of linear change and deceleration. Estimation of the component of projected change potentially amenable to intervention, future health targets and the projected impact on type 2 diabetes, were done.

**Results:**

Obesity prevalence in Quebec is projected to rise steadily from 2013 to 2030 in both men (from 18.0-19.4% to 22.2-30.4%) and women (from 15.5-16.3% to 18.2-22.4%). Corresponding projected numbers of obese individuals are (579,000-625,000 to 790,000-1,084,000) in men and (514,000-543,000 to 661,000-816,000) in women. These projected increases are found to be primarily an ‘epidemiologic’ rather than ‘demographic’ phenomenon and thus potentially amenable to public health intervention. Assessment of obesity targets for 2020 illustrates the necessity of using projected rather than current prevalence; for example a targeted 2% drop in obesity prevalence relative to 2013 translates into a 3.6-5.4% drop relative to 2020 projected levels. Type 2 diabetes is projected to increase from 6.9% to 9.2-10.1% in men and from 5.7% to 7.1-7.5% in women, from 2011–2012 to 2030. A substantial proportion of this change (25-44% for men, and 27-43% for women) is attributable to the changing BMI distribution.

**Conclusions:**

Obesity in Quebec is projected to increase and should therefore continue to be a public health priority. Application of projections to estimate the proportion of change potentially amenable to intervention, feasible health targets, and future chronic disease prevalence are demonstrated. Projection analyses have limitations, but represent a pertinent tool for public health planning.

**Electronic supplementary material:**

The online version of this article (doi:10.1186/1471-2458-14-996) contains supplementary material, which is available to authorized users.

## Background

The prevalence of obesity in Quebec (the second most populous province of Canada, with an estimated population of 8,067,319 in 2013) has seen a continuous increase over at least the past 2.5 decades [1, 2], mirroring trends in developed countries around the world [3]. From 1987 to 2012 for example, the prevalence of (self-report) obesity in Quebec adults more than doubled in value from ≈ 8% to 17%. These trends indicate a growing health and economic burden, as elevated BMI is associated with a range of co-morbidities and increased mortality [4–7].

Projection analyses of obesity have been done with increasing frequency in recognition of the need to estimate the future magnitude of this public health issue, and thus to plan health services, programs and interventions [8–13]. Projection studies however are not standard surveillance tools used by public health officers, and are largely the purview of university research scientists who may have different aims and perspectives. The objective of the current study is thus to provide projection analyses of BMI prevalence for the Quebec adult population, with the aim of informing and supporting public health planning. Short-term (2012–2019) projections using simple linear regression [12] represent the only other known projection study of Quebec obesity trends. Our study provides long-term (2013–2030) projections using a weighted compositional approach that overcomes methodological shortcomings of simple linear regression that may lead to bias and inaccuracy [10].

One issue that may limit the adoption of projection analyses for public health planning is that public health professionals may not be aware of the ways in which projections can be applied to estimate outcomes more directly related to policy and programs. To address this issue, three methodological techniques by which BMI prevalence projections can be translated into more concrete measures for health planning are demonstrated: (1) the separation of the projected time trends into demographic and epidemiologic components and thus estimation of the component potentially amenable to intervention, (2) use of projections in the planning of health targets including metrics that measure the difficulty of achieving targets as well as the consequences of not achieving them, and (3) the estimation of the projected impact on chronic disease using type 2 diabetes as an example, including estimation of the proportion of diabetes prevalence change potentially amenable to intervention.

Finally, projections may be perceived as technical and abstract mathematical constructs that are far removed from the multi-faceted (e.g. social, cultural, and technological) and complex nature of real-world public health issues. Thus we also provide a detailed discussion of the major limits and assumptions of the scenarios that underlie the BMI projections and suggest ways in which the results can be interpreted.

## Methods

### Data sources and variables

BMI prevalence time series were constructed from available cross-sectional surveys that measured self-report height and weight, and were representative of the Quebec population of adults 18 years of age and over. All surveys excluded the northern health regions of Nunavik and Terre-Cries-de-la-Baie-James. Sixteen independent survey cycles spanning the years from 1987 to 2012 were identified. These included four survey types: the Quebec Health Survey or ESQ (1987) [14] and the Quebec Health and Social Survey or ESS (1992–1993, 1998) [15] which were conducted by the Quebec Statistics Institute, and the National Population Health Survey or NPHS (1994–1995, 1996–1997, 1998–1999) [16] and the Canadian Community Health Survey or CCHS (2000–2001, 2002, 2003, 2005, 2007, 2008, 2009, 2010, 2011, 2012) [17, 18] which were conducted by Statistics Canada. A description of each survey cycle, including name of survey, corresponding year, and sample size is provided in Table 1. Survey data and values were extracted from master files for all cycles.

**Table 1:** **Description of the 16 survey cycles used to construct the BMI time series**

	Year	Name of survey	Abbr.	Sample size**	Source
1	1987	Quebec Health Survey	ESQ	17,494	Quebec Statistics Institute
2	1992-1993	Quebec Health and Social Survey	ESS	21,563	Quebec Statistics Institute
3	1994-1995	National Population Health Survey	NPHS	2,304	Statistics Canada
4	1996-1997	National Population Health Survey	NPHS	2,218	Quebec Statistics Institute
5	1998	Quebec Health and Social Survey	ESS	18,779	Statistics Canada
6	1998-1999	National Population Health Survey	NPHS	2,326	Statistics Canada
7	2000-2001	Canadian Community Health Survey	CCHS	20,161	Statistics Canada
8	2002	Canadian Community Health Survey*	CCHS	4,581	Statistics Canada
9	2003	Canadian Community Health Survey	CCHS	24,789	Statistics Canada
10	2005	Canadian Community Health Survey	CCHS	26,633	Statistics Canada
11	2007	Canadian Community Health Survey	CCHS	10,802	Statistics Canada
12	2008	Canadian Community Health Survey	CCHS	10,735	Statistics Canada
13	2009	Canadian Community Health Survey	CCHS	10,216	Statistics Canada
14	2010	Canadian Community Health Survey	CCHS	10,214	Statistics Canada
15	2011	Canadian Community Health Survey	CCHS	10,548	Statistics Canada
16	2012	Canadian Community Health Survey	CCHS	10,588	Statistics Canada

*Corresponds to Cycle 1.2: Mental Health and Wellbeing.

**Sample Size corresponds to individuals 18+ years of age, from Quebec, with valid BMI.

For each survey cycle, individual-level BMI was calculated as the weight in kilograms divided by the square of the height in meters; women that were pregnant at the time of the survey were excluded. Continuous BMI values were then classified into 4 categories according to standard WHO categorization [6]: Underweight (BMI < 18.5), Normal (18.5 ≤ BMI < 25), Overweight (25 ≤ BMI < 30), and Obese (BMI ≥ 30). The prevalence of each BMI category was calculated for 4 categories of age (18–24, 25–44, 45–64, 65+) and by sex. Variances and covariances were estimated using bootstrap (estimations from NPHS and CCHS) or Taylor linearization (estimations from ESQ and ESS) methods that accounted for the complex design of the surveys [19–21]. A total of 128 age, sex and time point combinations were available for analysis. Of these, 12 combinations were excluded: six due to missingness in the BMI variable that exceeded 10% [22] and six due to their imprecision or small cell counts and the diffusion rules of the statistical agencies that managed the surveys [23, 24].

Historical time series of the Quebec population by age and sex, as well as official population projections used in BMI projections and secondary analyses, were obtained from the Quebec Statistics Institute [25]. For projections of the impact of BMI on type 2 diabetes (T2D), estimates of the prevalence of T2D by BMI category, age group and sex were obtained from the CCHS, Cycle 2011–2012.

### Projections of prevalence and numbers of individuals by BMI category, 2013–2030

Regression is a frequently used approach in BMI and obesity projections [8–10, 12], and represents an accessible and intuitive way to model and project trends in cross-sectional survey time series. Of the many regression approaches that have been used, we consider the compositional approach [10, 26] to be the most statistically valid for the projection of BMI prevalence. In this approach, the set of prevalences corresponding to the four BMI (*i*) categories, for specified age (*j*) and sex (*k*), , are transformed and projected together, accounting for the multinomial nature of . Proper modeling of the above characteristics in prevalence time series is essential to reduce bias and inaccuracy that can become sizeable especially for longer term projection horizons [10]. We have further extended the compositional approach to account for the heterogeneity in survey variance structure, by including a weighting effect of the covariance matrix of  from each survey cycle. Prediction intervals to assess statistical variability, and goodness of fit were also estimated. Technical details of the method including sensitivity analyses can be found in Additional files 1 and 2.

Two scenarios are projected. In the first scenario, linear time trends are fitted to the transformed prevalence, and are assumed to continue into the future; the assumption of linear trends is considered a default model that is most often used in the projection literature. In the second scenario, logarithmic trends are fitted and assumed to continue into the future. This model has been considered in recent projection analyses to account for possible future stabilization in US and European obesity trends [8, 27]. These two scenarios were considered reasonable based on the scientific literature. The ‘linear’ scenario is considered ‘pessimistic’ since it results in a roughly linear continuation of the increasing obesity prevalence observed in all age and sex categories, while the logarithmic model is considered an ‘optimistic’ or ‘deceleration’ scenario as it projects a future decrease in the rate of change of historical trends, leading to a diminution of the rate of obesity increase.

Corresponding projected numbers of individuals by BMI (*i*), age (*j*) and sex (*k*) are obtained by the product of projected prevalence and projected population estimates: *N*_*ijk*_(*t*) = *P*_*ijk*_(*t*)*n*_*jk*_(*t*), where *N*_*ijk*_(*t*) and *P*_*ijk*_(*t*) are the projected numbers and prevalence respectively, and *n*_*jk*_(*t*) represents the projected population by age and sex category. The age aggregated number of individuals *N*_*ik*_(*t*) is equal to the sum of numbers over all age groups: , while the age aggregated prevalence *P*_*ik*_(*t*) is equal to *N*_*ik*_(*t*) divided by the corresponding population: *P*_*ik*_(*t*) = *N*_*ik*_(*t*)/*n*_*k*_(*t*). *N*_*ik*_(*t*) can be interpreted as a measure of the absolute burden, while *P*_*ik*_(*t*) is a measure of the BMI distribution in the Quebec population. The principle results of the projection analysis thus comprise  and  which are the prevalence and number of individuals for the linear and deceleration scenarios respectively.

### Separation of epidemiologic and demographic contributions

Temporal change in the projected prevalence and number of individuals can be approximately decomposed into two components: a demographic effect which represents the contribution of changes in population age structure, and an epidemiologic effect which represents the contribution of changes in age- and sex-specific BMI prevalence which are independent of population age structure. This decomposition is informative as the epidemiologic effect can be interpreted as the component of change that is potentially amenable to intervention while the demographic effect (i.e. the effect of population aging) cannot in general be modified. In practice, any actual interventions are highly unlikely to eliminate the epidemiological effect completely, and so this effect represents a hypothetical maximum. The separation of the demographic effect from overall trends is especially pertinent in the case of Quebec (as well as other developed nations) in which there is a strong demographic shift to an older population age structure, which can drive a range of health issues.

The demographic contributions to age aggregated prevalence and numbers () are first estimated by holding age and sex specific BMI prevalences fixed at a reference level, *P*_*ijk*_(*t*_*ref*_), while allowing only the population to evolve. The epidemiologic contribution is then estimated by subtracting the demographic effect from the overall trend and thus effectively represents the contribution attributable to other risk factors. Thus  and .  is of particular interest as it can be interpreted as the number of cases of obesity that are potentially amenable to interventions that target the population BMI distribution. For this reason,  will be termed the number of ‘avoidable cases’.

In the current study, demographic and epidemiologic changes in obesity trends are estimated relative to two reference times: (1) *t*_*ref*_ = 2011 − 2012 in order to estimate the proportion of future projected changes in obesity prevalence potentially amenable to intervention (the aggregate of two cycles is implemented here to ensure adequate precision in the prevalence estimates), and (2) *t*_*ref*_ = 1987 in order to estimate obesity prevalence under the hypothetical scenario that the BMI distribution ‘returns’ to past levels.

### Planning of future health targets

The application of projections to the planning of health targets will be shown by the quantitative assessment of three hypothetical targets for 2020 (using a planning date of 2013) in the context of projected trends. The hypothetical future targets comprise: (1) a 2% reduction in prevalence relative to 2013 estimated values, (2) a target that holds obesity prevalence constant and equal to 2013 values and (3) a 2% reduction in obesity prevalence relative to 2020 projected values.

Three quantitative measures will be applied in the assessments: (1) The gap or difference between target and projected prevalence, *ΔP*_*TP*_, which measures the difficulty of achieving a future target, (2) the average annual rate of change in prevalence (*AAC*_*TP*_) to achieve a target, which is a measure of the yearly effort required to change projected trends; and (3) the difference between target and projected numbers of individuals *ΔN*_*TP*_, which represents a measure of the consequence in terms of population burden, of not achieving the targets.

### Projected impact on type 2 diabetes

The expected increase in the prevalence of numerous co-morbidities (type 2 diabetes, hypertension, incapacity, etc.) represents a primary public health concern associated with rising obesity levels [28, 29]. Projection analyses of BMI can be applied to estimate the magnitude of increase in associated co-morbidity prevalence, which will be demonstrated using type 2 diabetes (T2D). The projected number of cases of T2D by BMI, age and sex (*η*_*ijk*_(*t*)), is estimated as the product of the estimated prevalence of T2D in each BMI, age and sex category (*π*_*ijk*_) with the corresponding projected numbers of individuals: *η*_*ijk*_(*t*) = *π*_*ijk*_ × *N*_*ijk*_(*t*). Aggregation over BMI and age categories is then done to obtain total projected numbers of cases  and prevalence  by sex, where *n*_*k*_(*t*) represents the projected population by sex. A basic assumption in this estimation is that the BMI, age and sex specific prevalence of T2D (*π*_*ijk*_) is held fixed and not in itself projected; for the present analysis *π*_*ijk*_ is estimated from the pooled 2011–2012 CCHS cycles. *η*_*k*_(*t*) and *π*_*k*_(*t*) thus represent the projected chronic disease numbers and prevalence respectively where the age, sex and BMI specific T2D prevalence profile remains constant but the BMI distribution and population evolve, and can be interpreted as a measure of the projected combined impact of BMI epidemiology and population demographics. Estimation of the epidemiologic contribution to *η*_*k*_(*t*) and *π*_*k*_(*t*) can be done as before (Additional file 3, Section A3.1 describes this calculation in greater detail).

## Results and discussion

### Projections of prevalence and numbers by BMI category, 2013–2030

**Figure 1:**
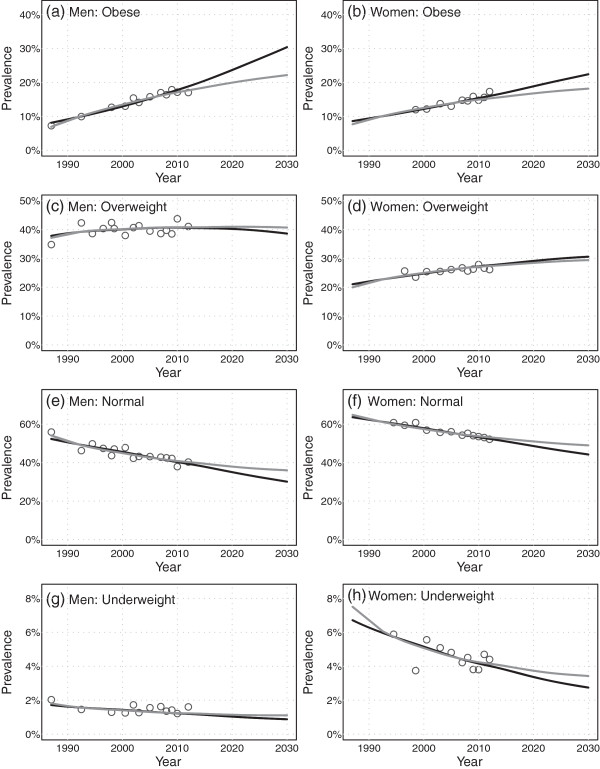
**Projections (2013 to 2030) of age-aggregated prevalence by BMI category, for men and women.** The linear scenario is indicated by the black line, the deceleration scenario is indicated by the gray line, and the historical BMI time series data are indicated by the open circles.

Projection results showing fitted and projected age aggregated prevalence are shown in Figure 1a to h for men and women. The ‘pessimistic’ linear scenario  is represented by the black line and the ‘optimistic’ deceleration scenario  is represented by the gray line; the range of possible future outcomes can be interpreted as falling between the span of the two scenarios [30, 31]. Values representing age aggregated survey time series data are indicated by the circles. Projected prevalence values are listed in Table 2a and b for the years 2013, 2020 and 2030.

As can be seen, obesity prevalence is projected to rise steadily from 2013 to 2030 for both scenarios in both men (18.0-19.4% to 22.2-30.4%) and women (15.5-16.3% to 18.2-22.4%). Here, and for the results that follow, a reported range in values corresponds to the projection results of the deceleration (optimistic) and linear (pessimistic) scenarios respectively. These projections translate to 23-57% and 17-37% proportional increases in prevalence for men and women respectively. Prevalence of overweight (Figure 1c and d) are projected to remain fairly stable for men (40.8-40.6% to 40.7-38.6%) and to increase slowly for women before also stabilizing (27.5-27.8% to 29.4-30.6%). Normal weight prevalence (Figure 1e and f) is projected to decrease steadily in both men (40.0-38.9% to 36.0-30.1%) and women (52.9-51.9% to 49.0-44.2%). Finally the prevalence of underweight (Figure 1g and h) is projected to decrease in both men (1.2-1.2% to 1.1-0.9%) and women (4.1-3.9% to 3.4-2.7%), though the statistical variability for this category is high.

**Table 2:** **Projected prevalence by BMI class for the years 2013, 2020 and 2030 for (a) Men and (b) Women; pairs of values represent the range spanned by the deceleration and linear scenarios respectively**

Men: Projected Prevalence by BMI Class				
Year	Underweight	Normal	Overweight	Obese
2013	1.2% - 1.2%	40.0% - 38.9%	40.8% - 40.6%	18.0% - 19.4%
2020	1.1% - 1.0%	37.9% - 35.1%	41.0% - 40.2%	19.9% - 23.7%
2030	1.1% - 0.9%	36.0% - 30.1%	40.7% - 38.6%	22.2% - 30.4%
**Women: Projected Prevalence by BMI Class**				
**Year**	**Underweight**	**Normal**	**Overweight**	**Obese**
2013	4.1% - 3.9%	52.9% - 51.9%	27.5% - 27.8%	15.5% - 16.3%
2020	3.7% - 3.4%	51.0% - 48.6%	28.4% - 29.1%	16.8% - 18.9%
2030	3.4% - 2.7%	49.0% - 44.2%	29.4% - 30.6%	18.2% - 22.4%

Fitted and projected trends are quite similar between men and women though absolute levels of prevalence for obesity and overweight are higher for men vs. women, and absolute levels of prevalence for normal weight and underweight are lower for men vs. women. Trends in the two scenarios are also similar, with absolute rates of change being attenuated in the deceleration scenario as compared with the linear scenario. These trends are found to be similar and consistent across all age categories (Additional file 4).

**Figure 2:**
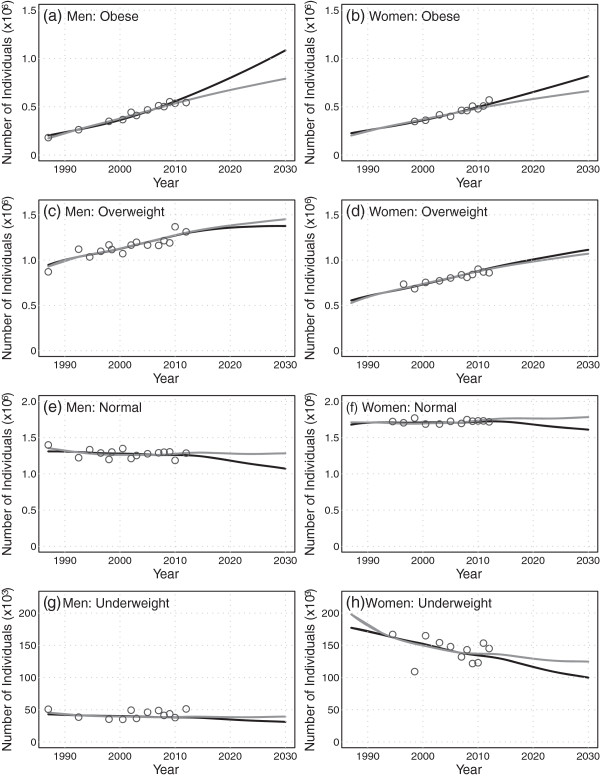
**Projections (2013 to 2030) of age-aggregated numbers of individuals by BMI category, for men and women.** The linear scenario is indicated by the black line, the deceleration scenario is indicated by the gray line, and the historical BMI time series data are indicated by the open circles.

Projected age aggregated numbers of individuals,  and , are shown in Figure 2a and b for men and women. Projected values are also listed in Table 3a and b for the years 2013, 2020 and 2030. The projected trends in the number of individuals are similar to those for prevalence with an additional increasing trend due to the overall increase in total population projected from 2013 to 2030 (Column 2 of Table 3a and b). In particular, the number of obese individuals is projected to rise steadily from 2013 to 2030 for both scenarios and for both men (579,000-625,000 to 790,000-1,084,000) and women (514,000-543,000 to 661,000-816,000), while the number of overweight individuals (Figure 2c and d) are projected to increase slowly for men (1,315,000-1,308,000 to 1,451,000-1,376,000) and for women (913,000-923,000 to 1,071,000-1,114,000).

**Table 3:** **Projected numbers of individuals by BMI class for the years 2013, 2020 and 2030 for (a) Men and (b) Women; pairs of values represent the range spanned by the deceleration and linear scenarios respectively**

	Men: Projected Numbers by BMI Class				
Year	Adult Population	Underweight	Normal	Overweight	Obese
2013	3,225,000	39,000 – 38,000	1,291,000 – 1,254,000	1,315,000 – 1,308,000	579,000 – 625,000
2020	3,376,000	38,000 – 35,000	1,281,000 – 1,183,000	1,383,000 – 1,358,000	673,000 – 799,000
2030	3,563,000	39,000 – 31,000	1,283,000 – 1,072,000	1,451,000 – 1,376,000	790,000 – 1,084,000
	**Women: Projected Numbers by BMI Class**				
**Year**	**Adult Population**	**Underweight**	**Normal**	**Overweight**	**Obese**
2013	3,321,000	136,000 – 131,000	1,757,000 – 1,682,000	913,000 – 923,000	514,000 – 543,000
2020	3,459,000	129,000 – 117,000	1,765,000 – 1,682,000	984,000 – 1,008,000	580,000 – 652,000
2030	3,640,000	125,000 – 100,000	1,783,000 – 1,610,000	1,071,000 – 1,114,000	661,000 – 816,000

The projected substantial and continued increase in obesity prevalence for Quebec are in line with other projection studies done for Canada [12, 32, 33], the USA [8, 34], UK [10, 35, 36] and Australia [36] for a projection horizon up to 20 years and using a range of methods. In particular, projected prevalences are similar to short-term (2012–2019) projection results made using simple linear regression on CCHS data [12], the only other known projection study of Quebec BMI trends. These overall trends in prevalence indicate that the population BMI distribution is shifting to the right as well as supporting observations from other studies that indicate a widening and increasing skew [10, 33, 36, 37].

Visual assessments indicate good fit of both linear and log models to measured data in both age aggregated and age specific results and projected trends are seen to be smooth extensions of clear historical trends (cf. Figure 1 and Additional file 4). Goodness of fit for obesity prevalence is further confirmed using the *R*^2^ statistic (adapted for GLS) which yields values ranging from 0.44-0.97 for age and sex specific trends (Additional file 2). Prediction intervals (shown in Additional file 2: Figure A2) indicate that the fitted models are statistically stable, though these intervals should not be interpreted as uncertainty bounds **(cf Discussion)**. Finally, sensitivity analyses using a recent (2000–2012) subset of the measured data show that projected trends are robust (Additional file 2: Figure A2).

Overall, the projection analyses indicate that obesity will represent a growing health burden on the Quebec population, and public health challenge. These results are statistically robust; however they are based on the chosen scenarios and on the assumption that current trends will continue into the future.

### Separation of epidemiologic and demographic contributions

**Figure 3:**
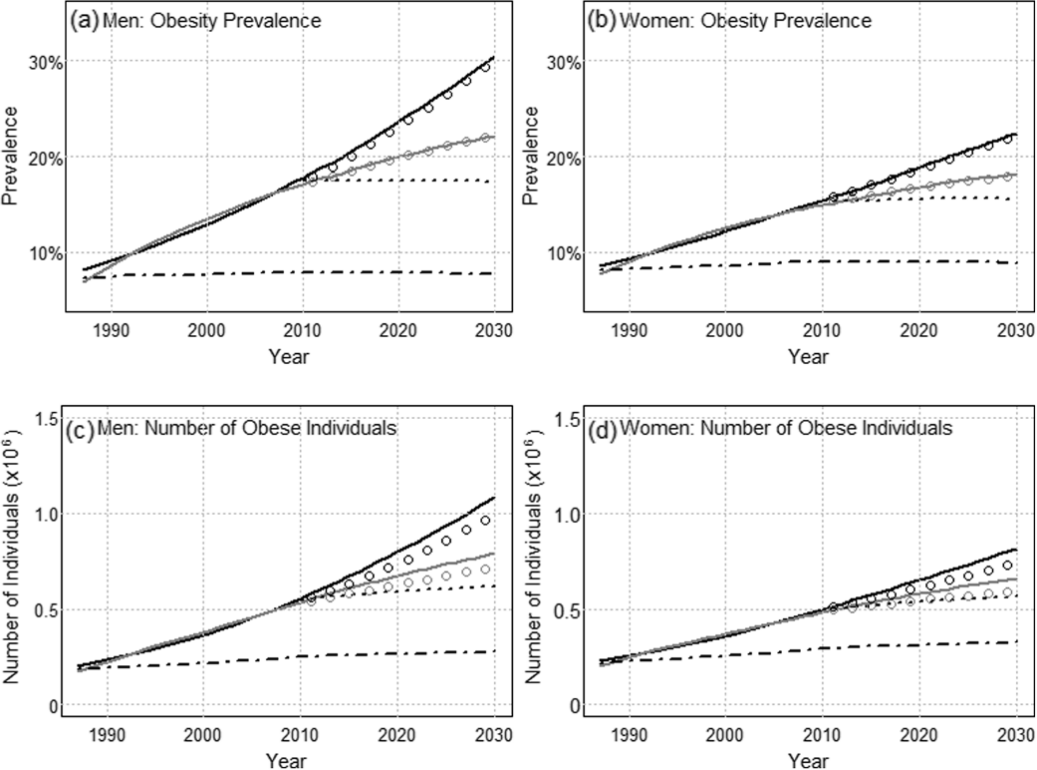
**Separation of epidemiologic and demographic contributions to the projected change in obesity prevalence and numbers of individuals, for men and women.** The linear scenario is indicated by the black line and the deceleration scenario is indicated by the gray line. The demographic projection using 2011–2012 as reference is indicated by the dotted line; the corresponding epidemiologic projections are indicated by the black and gray circles. The demographic projection using 1987 as reference is indicated by the dashed line.

Estimated demographic and epidemiologic contributions to projected change are shown in Figure 3. The demographic effect in prevalence , where age and sex specific prevalences have been fixed at 2011–2012 values are shown by the dotted lines in Figure 3a and b for men and women respectively. It can be seen that  appears flat and unchanged from the 2011–2012 obesity prevalence, indicating that demographic changes plays virtually no role in the projected obesity trends. As a result, the projected increase in obesity prevalence is almost purely an epidemiologic phenomenon and thus is in principle potentially amenable to intervention. This is further shown by plots of the epidemiologic effect  which are represented as black and gray circles; the points are closely coincident with the corresponding linear and deceleration scenarios.

The demographic effect is expected to contribute slightly to the number of obese individuals (Figure 3c and d); this is due to the dependence of  on not only the age structure but the total population size which is projected to increase (Table 3). As a result, epidemiologic effects, shown by the black and gray circles, account for most but not all of the projected increase in obesity numbers. Calculated values of  for the two scenarios indicate approximately 76,000-202,000 and 12,000-84,000 additional obese individuals will accrue by 2020 for men and women respectively, due to changes in BMI epidemiology; these represent the number of avoidable cases of obesity as defined earlier. These numbers increase to 163,000-456,000 and 70,000-225,000 by 2030 for men and women respectively.

The demographic effect in prevalence , where age and sex specific prevalences have been fixed at 1987 values are shown by the dashed lines in Figure 3.  remains virtually unchanged from the fitted 1987 obesity prevalence; the epidemiologic contributions are not shown but follow the total projected trends closely. This result suggests that if the population BMI distribution could somehow be returned to that of 1987, the overall obesity prevalence would return to 1987 levels as well, in spite of changes in population age structure that have occurred since then.

 is projected to increase slightly in both men and women. Calculated values of  represent the number of avoidable cases of obesity should the BMI distribution return to that of 1987. By 2013, the first projection year, it is estimated that approximately 321,000-367,000 and 203,000-232,000 obese men and women were accrued due to changes in the BMI distribution relative to 1987. In 2030 the number of avoidable cases of obesity is projected to reach 509,345-803,000 and 322,000-477,000 men and women, relative to 1987.

In the present study, it is shown that projected changes in obesity are almost completely an epidemiologic effect. Thus future increases in obesity prevalence and numbers could in principle be eliminated if increases in age specific obesity prevalence were to be halted. These analyses suggest that holding the population BMI distribution constant at 2011–2012 levels or ‘returning’ the BMI distribution to 1987 levels could both be considered as concrete and beneficial intervention benchmarks.

### Planning of future health targets

**Figure 4:**
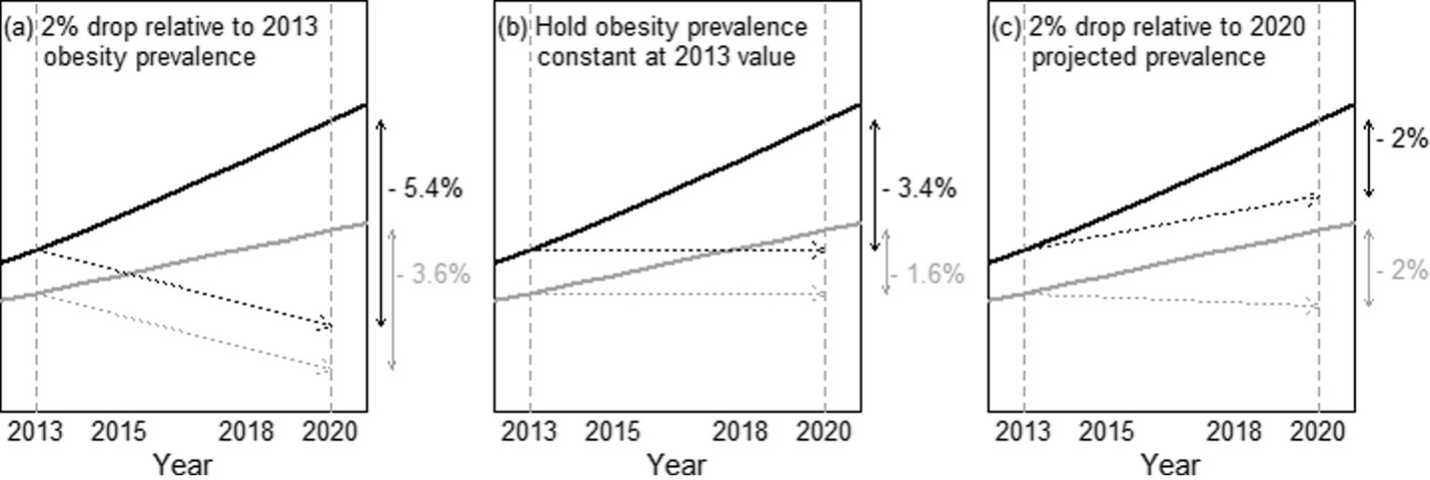
**Three hypothetical future obesity targets are shown, superimposed on projected trends.** In each graph, the black line indicates the linear scenario and the black dotted arrow indicates the corresponding target relative to the projection. The gray line indicates the deceleration scenario and the gray dotted arrow indicates the corresponding target. Numbers to the right of each graph indicate the difference between targeted and projected prevalence (*ΔP*_*TP*_) for the linear (black) and deceleration (gray) scenarios respectively.

Figure 4a-c shows the three hypothetical obesity prevalence targets for the time period 2013–2020, with projections over this period from both scenarios superimposed. In Figure 4a, a targeted decrease in obesity prevalence of 2% for 2020 relative to 2013 estimated levels is compared to projected trends. Achievement of this target would require a substantial deviation (*ΔP*_*TP*_ = − 3.6 to − 5.4%) relative to projections and an average annual change (*AAC*_*TP*_) of −0.52 to −0.77%; the number of obese individuals (*ΔN*_*TP*_) would be reduced by an estimated 249,000-370,000. Figure 4b shows a more modest target that aims to hold obesity prevalence constant at 2013 levels. Achievement of this target would nevertheless still require a deviation *ΔP*_*TP*_ = −1.6 to −3.4% from projected trends, and an *AAC*_*TP*_ of −0.23 to −0.49%; an estimated (*ΔN*_*TP*_) = 112,000 − 233,000 obese individuals could be avoided. Finally, Figure 4c shows a target based on a 2% decrease in obesity prevalence relative to projected values for 2020. In this case, the required deviation from projected trends is specified by the target itself to be at *ΔP*_*TP*_ = 2% and is associated with an *AAC*_*TP*_ of −0.29% per year. The projected 2020 obesity prevalence should this target be achieved is 16.3-19.2% and includes the possibility of an increase in prevalence from 2013. Achieving this target would reduce the projected number of obese individuals by an estimated 137,000 for both scenarios.

The application of quantitative techniques, including the use of projections, has been advocated in the health policy literature [38, 39] for the planning of feasible health targets. The estimation of the gap between target and projected prevalence (*ΔP*_*TP*_) and the average annual rate of change required (*AAC*_*TP*_) in the current study demonstrates the necessity to assess targets based on future projected prevalence corresponding to the target date, rather than prevalence levels at the start or planning date. For example, the targeted 2% reduction in obesity prevalence relative to 2013 shown in Figure 4a is representative of the kind of targets implemented in public health programs worldwide, where efforts are typically announced to improve on current health levels [40, 41]. However as shown, the difficulty of achieving this target becomes greatly amplified (to a 3.6-5.4% prevalence reduction), when compared with projected trends, which likely represents a more accurate and realistic measure. Use of the projected prevalence as a point of reference thus correctly accounts for the additional effect of time trends that need to be countered in order to achieve targets. Estimation of the consequence of not achieving targets is considered an essential element in target planning and evaluation [42]; projections permit estimation of the number of avoidable cases of obesity, *ΔN*_*TP*_, which represents one such measure. More generally, the planning of feasible health targets could be further improved by the integration of quantitative projections with the estimated impact of available intervention programmes, an area of ongoing research.

### Projected impact on type 2 diabetes

**Figure 5:**
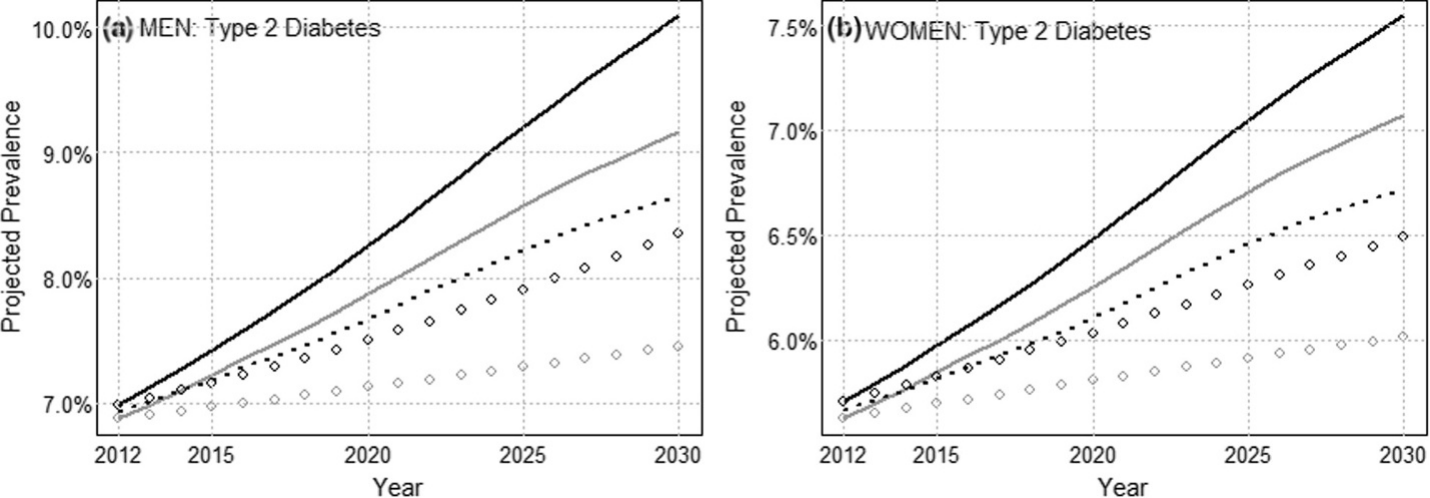
**Projections of the impact of BMI on type 2 diabetes prevalence.** The linear scenario is indicated by the black line and the deceleration scenario is indicated by the gray line. The demographic projection using 2011–2012 as reference is indicated by the dotted line; the corresponding epidemiologic projections are indicated by the black and gray circles.

The estimated BMI and age specific T2D prevalences (*π*_*ijk*_) for 2011–2012 show marked increasing trends with both BMI and age in men and women (Additional file 3: Figure A3.1). Projections of age aggregated T2D prevalence are shown in Figure 5 for (a) men and (b) women. T2D prevalence in adult men, estimated at ≈ 6.9% in 2011–2012, is projected to increase to 7.9-8.3% by 2020, and to 9.2-10.1% by 2030, due to combined BMI and demographic effects. T2D prevalence in women, estimated at ≈ 5.7% in 2011–2012, is projected to increase to 6.3-6.5% by 2020, and to 7.1-7.5% by 2030.

The black and gray circles represent the epidemiologic contribution (since 2011–2012) estimated by the two projection scenarios. In particular, for men the epidemiologic component of change  is estimated to be ≈ 0.6-1.4% in 2030, or to contribute ≈ 25-44% of the total projected increase in T2D prevalence relative to 2011–2012. This corresponds to ≈ 20,000-49,000 avoidable cases. For women  is estimated to be ≈ 0.4-0.8%, or to contribute ≈ 27-43% of the total projected increase, corresponding to ≈ 14,000-29,000 avoidable cases. Results thus indicate that increasing BMI and aging of the Quebec population will likely combine to drive projected future increases in overall T2D prevalence. Estimation of the epidemiologic component of change further shows that a substantial component of the projected change will in principle be amenable to interventions that act on the BMI distribution of the population.

The projection analyses of T2D make the basic assumption that the prevalence of T2D by age, sex and BMI categories will remain constant in time. In reality this assumption will not hold due to temporal change in T2D epidemiology as well as the dependence of T2D prevalence at a given moment in time on the cumulative effect of past trends. Nevertheless, this kind of projection provides useful insight into the evolution of disease burden for public health planning, and similar estimations have been implemented in other studies [35, 43]. For example, the projection estimates can be interpreted as a baseline or counterfactual scenarios on which further hypotheses or scenarios (related to specified changes to the T2D prevalence profile) could be based. Alternatively they could be interpreted as a hypothetical scenario where the current T2D prevalence profile were to be applied to a future population (with corresponding future BMI and population structure). An internal validation study in fact suggests that *π*_*ijk*_ is fairly stable in time, and that the projections of T2D may represent reasonable though slightly conservative future estimates (data not shown).

### Limitations and interpretation of projection results

Examination and discussion of the major assumptions, limitations as well as strengths underlying the projection analyses are next provided, with the aim of guiding their interpretation and use for public health planning.

### Scenarios vs. projections

A key concept in the interpretation and evaluation of projection analyses is that they can be thought of as quantitative expressions of underlying qualitative scenarios. Scenarios are non-quantitative, non-probabilistic, plausible portraits of alternative futures; they can be considered as ensembles of diverse assumptions, and are typically formulated by expert opinion [31, 44, 45]. Thus while projection analyses are themselves mathematically rigorous, their validity depends in fact on the largely qualitative choice of underlying scenario. In the current study, scenarios consisted of the linear and deceleration models which are the predominant models in the obesity projection literature and were also deemed most plausible; as well, they appeared reasonable based on the observed trends in the historical data. The accuracy of these scenario choices with respect to the actual future is inherently unquantifiable however; a public health planner may either agree or disagree with the scenarios chosen, and this will determine the perceived accuracy or reliability of the results.

### Uncertainty and prediction intervals

Prediction intervals are known to greatly underestimate the actual error when applied to projection analyses [46, 47]. This is because prediction intervals account for the statistical error due to data variability and estimation, but do not account for the error in scenario choice which can be thought of as model misspecification. For this reason, the values spanned by the range of pertinent scenarios are often used as a way to reasonably gauge the uncertainty in projections [30, 31]. In the current study for example, while prediction intervals show that the projection models are statistically stable, the linear and deceleration scenarios are used as reasonable bounds on actual future BMI trends. Overall it should be emphasized that projection results should not be interpreted as infallible forecasts, but rather as estimates whose uncertainty depends on both statistical variability and on the perceived validity of underlying scenarios.

### The extrapolation assumption

All projections that make use of historical data make the ‘extrapolation assumption’ that past trends in these data will continue into the future. In the current study time trends in BMI are modelled using a period (or calendar time) effect to essentially represent in an aggregate manner, the effect of all causative factors over time (including the effects of lifestyle habits such as diet and exercise, medical and activity-reducing technology, food prices etc.). The assumption that these trends will continue into the future is not unreasonable given that population BMI is affected by a diversity of factors whose aggregate effect is likely to vary smoothly and slowly in the future, as they have done so in the past. In light of the ‘extrapolation assumption’, projection analyses can be interpreted as being baseline trends that could represent upper/lower or pessimistic/optimistic limits to actual trends, depending on shifts in external causative factors that might be hypothesized to occur. Alternatively, deviations in actual trends from projections could be retrospectively interpreted as evidence that changes in causative effects have occurred and in particular, provide evidence for the efficacy of interventions [48, 49].

### Cohort effects

Cohort effects were not modeled explicitly in the current projection analysis as the restricted length relative to human lifetimes, and irregular spacing of the measured time series data prevented reliable estimation for projection purposes. The error induced by the absence of a cohort effect in projections is not expected to be substantial however. Age-period-cohort analyses have shown that period effects (as represented by the time variable in our regression model) represent the dominant contribution to obesity trends [50–52]. One interpretation of the current projection scenarios is that they represent a neutral middle ground between the positive cohort effects detected by some studies [50, 53], and the negative effects detected by others [54]. Alternatively, if recent Quebec cohorts are believed to have increased obesity prevalence, obesity projection results can be interpreted as conservative underestimates; on the other hand, if recent cohorts are believed to have reduced obesity due to Quebec programs that have targeted children and youth for example [55], the projections could be considered pessimistic overestimates.

### Interpretation of Epidemiologic Effects

In our analysis we present a decomposition of demographic and epidemiologic effects. It should be noted that our interpretation of demographic effects is restricted to variations in the BMI of sex and age groups in the adult Québec population. As well, there are other largely unalterable biological factors not taken into account in our analysis which may influence BMI (e.g., hormonal factors, genetics) [56]. It is furthermore unknown to what extent the epidemiologic effects are truly amenable due to the complex interaction of biological, behavioral and environmental factors on individual-level body weight [56]. Thus the epidemiological effect should be interpreted as an approximate and hypothetical upper limit to what might be potentially amenable to public health intervention.

### Self-report BMI

Self-report BMI is known to underestimate measured BMI [57], though the two measures are highly correlated [58, 59]. The use of self-report BMI in the present study, reflects a limitation in the available data, as Quebec-level surveys with measured BMI do not exist in sufficient numbers to form time series that can be projected reliably. Correction equations for self-report BMI have been proposed [60, 61]. However as the magnitude of the correction is known to vary with both time [62] and survey-specific characteristics [63], the accuracy of these equations outside of the particular survey cycles for which they were developed is uncertain [62, 64]. Thus it was felt that application of a global correction factor over the diverse surveys that comprise the historical time series could result in additional random as well as systematic error in both the BMI values and their time trend. The only published estimate of the magnitude of self-report bias in Quebec is from the 2008 CCHS, where self-report obesity prevalence in adults (18+) was found to underestimate measured prevalence by 8.8% [2]. This value is higher than Canada-wide estimates of 7.6% for men and 7.2% for women aged 18–79, measured from the same data [63]. A previous Canadian study found that self-report bias had increased from 4% in 1986–1992 to 8% in 2005 (for adults aged 18–74 years), suggesting that self-report bias was increasing with time in Canadian survey data [62]. However a subsequent study did not detect a statistically significant change between 2005 and 2008 estimates [63]. Data taken from disparate Canadian surveys [65] suggest that the magnitude of self-report bias has at least been maintained since 2008. In light of the bias attributable to self-report BMI, the results of the current projection analysis should be interpreted as conservative underestimates of the actual underlying measured BMI trends. Overall, projection results indicate a continual and alarming rise in future self-report obesity that almost certainly reflects higher absolute levels and greater increases in actual measured obesity.

### Strengths of the current analysis

Within the framework of the chosen scenarios, the current projection analyses have many strengths. These include historical time series constructed from very large datasets comprising sixteen cycles from nationally representative surveys and sampling from a total of 203,951 individuals. The use of weighted compositional regression to model and project time trends is considered to be the most rigorous regression-based approach to fit time series of multi-category prevalence, and includes a weighting matrix that accounts for variance heterogeneity in the surveys as well as the covariances between BMI categories. Projection results show goodness of fit to clear and pronounced trends in the historical data, while prediction intervals and sensitivity analyses show that the results are statistically stable and robust.

## Conclusions

This study represents the first long-range projection analysis of the future obesity prevalence and numbers of individuals for the province of Quebec. Obesity prevalence is projected to continue rising over the next 18 years should past trends continue, reflecting a population BMI distribution that continues to shift to the right, toward higher values. Thus obesity is expected to continue to be a priority for public health policy and programs. Three secondary analyses have been presented that further illustrate the utility and pertinence of projection analyses to public health planning: (1) Decomposition of projected trends shows that increases in total prevalence and numbers of obesity in Quebec adults is primarily an ‘epidemiologic’ rather than ‘demographic’ effect and thus potentially amenable to public health interventions acting on the population BMI distribution, (2) Assessments of hypothetical obesity targets planned for 2020 illustrate the necessity of using projected rather than current prevalence levels to estimate the difficulty of achieving future targets, (3) Estimation of the projected impact on type 2 diabetes prevalence shows that while population aging is the principal driving force, a substantial component of the anticipated increase in type 2 diabetes prevalence and numbers of cases is potentially amenable to intervention.

Further continued dialogue with public health planners may lead to more refined models tailored to answer specific hypotheses or questions concerning public health challenges associated with obesity, such as the assessment of the impact of specific interventions or programs. More generally, it is hoped that this work will encourage and promote awareness and use of projections as a relevant and useful tool for public health planning.

## Electronic supplementary material

Additional file 1: **Description of the weighted compositional regression method for projection of multi-category BMI prevalence, including the method of estimation for prediction intervals, goodness of fit and sensitivity analyses.** (DOC 425 KB)

Additional file 2: **Prediction Intervals, Goodness of Fit and Sensitivity Analyses.** (DOC 442 KB)

Additional file 3: **Projection of type 2 diabetes: (1) separation of epidemiologic and demographic components, and (2) survey estimates of BMI, age and sex-specific prevalence of type 2 diabetes.** (DOC 238 KB)

Additional file 4: **Age-specific BMI prevalence projections.** (DOC 213 KB)

## Abbreviations

BMI: Body mass index
CCHS: Canadian Community Health Survey
ESQ: Quebec Health Survey (*Enquête Santé Québec*)
ESS: Quebec Health and Social Survey (*Enquête Sociale et de Santé*)
GLS: Generalised Least Squares
ISQ: Quebec Statistics Institute (*Institut de la Statistique du Québec*)
MSSS: Quebec Ministry of Health (*Ministère de la Santé et des Services Sociaux du Québec*)
NPHS: National Population Health Survey
T2D: Type 2 Diabetes.
