# Supplementary material for: Projection scenarios of body mass index (2013–2030) for Public Health Planning in Quebec
Source: BMC Public Health. 2014 Sep 25;14:996. doi: 10.1186/1471-2458-14-996 (PMC4196088; doi:10.1186/1471-2458-14-996)
Supplement: Supplementary file 1 — Additional file 1: Description of the weighted compositional regression method for projection of multi-category BMI prevalence, including the method of estimation for prediction intervals, goodness of fit and sensitivity analyses. (DOC 425 KB) [file 12889_2014_7135_MOESM1_ESM.doc]

Additional file **1: Description of the weighted compositional regression method for projection of multi-category BMI prevalence, including estimation of prediction intervals, goodness of fit and sensitivity analyses**

Each step of the weighted compositional projection method will be described in this Appendix. Vector and matrix notation will be used when possible for conciseness, and familiarity with standard multivariate regression and GLS (generalized least squares) estimation theory is presumed.

1. Construct measured BMI prevalence compositions and covariance matrix time series from survey data

Let
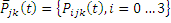
be the set of 4 BMI prevalences specific age group
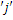
, sex
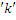
 and measured from survey timepoint
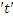
; the index
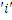
 corresponds to BMI category.
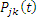
 is said to form a 4-dimensional ‘composition’ (Aitchison J 1986; Mills T 2009). Let
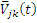
 be the 4x4 measured covariance matrix of
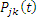
. The diagonal elements of
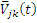
 represent the variances of the prevalences of each BMI category, while the off-diagonal elements represent the covariances between different BMI categories. In the current study, time series in
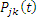
 and
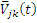
 comprise 16 survey cycles corresponding to timepoints
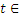
 (1987, 1992.5, 1994.5, 1996.5, 1998, 1998.5, 2000.5, 2002, 2003, 2005, 2007, 2008, 2009, 2010, 2011, 2012).

2. Transformation of 4-dimensional prevalence compositions to 3-dimensional real space

Statistical modelling or analyses of
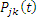
 must account for its particular numerical properties that include bounded support (each prevalence component is bounded between 0 and 1), positivity (prevalence components cannot be negative), summation constraint (
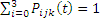
), and correlation between the components (including but not limited to, the effect of the common denominator). As shown by Aitchison (1986) and Aitchison and Shen (1980) (Aitchison J & Shen SM 1980; Aitchison J 1986), if
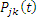
 has a multivariate logistic-normal distribution, the additive log-ratio transformation,
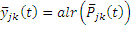
, can be applied to map
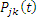
 to a 3-dimensional real space such that
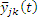
 is multivariate normal. The wide range of inference tools developed for multivariate normal data (including multivariate regression) can then be applied to
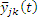
; results are then backtransformed to recover the original prevalence space. This compositional approach has distinct advantages over the use of simple linear regression; the latter method can result in biased projection estimates (Mills T 2009) as well as estimated values that violate the numerical properties described and are thus difficult to interpret.

The alr-transformation is shown below:


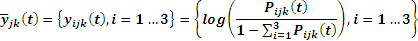


The prevalence component
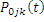
 is termed the ‘fill-up’ value and was assigned to the underweight BMI category; however the results of the analysis are in fact invariant to the choice of category for
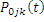
 (Aitchison J 1982).

3. Calculation of the transformed covariance matrices

Let
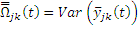
 denote the 3x3 covariance matrix of the transformed BMI composition corresponding to age category
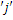
, sex
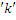
 and timepoint
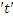
.
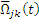
 is estimated from
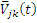
 using the delta method:


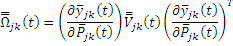


4. Projection analysis using weighted multivariate regression

Multivariate regression is used to fit and extrapolate trends in
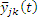
:


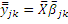


For the current study
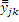
 is the 16x3 matrix representing the time series in each component of
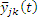
,
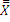
 is the 16x2 covariate matrix consisting of intercept and time columns, and
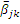
 is the 2x3 matrix of regression coefficients. To account for the covariance matrix unique to each survey timepoint, the preceding regression equation is reduced to a univariate form by ‘stacking’ the matrix terms (Jobson JD 1991):


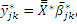


where
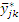
,
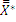
 and
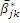
 are the corresponding 48x1, 48x6 and 6x1 stacked matrices.

The regression coefficients can then be estimated using the standard GLS estimator (Kutner M et al. 2005):


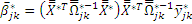
, where
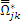
 is the ‘stacked’ covariance matrix that includes the transformed covariances for all 16 timepoints.

5. Estimation of projected prevalence

The projection estimate for future time
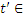
 (2013, 2014, … , 2030) is determined by extrapolation of the regression model, for example using the stacked notation:


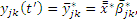


where
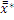
 is a 3x6 matrix that specifies the intercept and particular future time
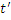
 for each component of
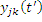
. Backtransformation
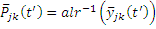
 is used to recover the projected BMI prevalences. The back transformation equations are shown below:

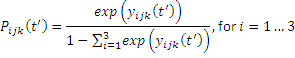


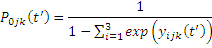


References

Aitchison J 1982. The Statistical Analysis of Compositional Data. J. R. Statist. Soc. B 44: 139-177.

Aitchison J 1986. The Statistical Analysis of Compositional Data. Chapman and Hall, London.

Aitchison J and Shen SM 1980. Logistic-normal distributions: Some properties and uses. Biometrika 67: 261.

Jobson JD 1991. Applied Multivariate Data Analysis: Regression and Experimental Design, Volume 1. Springer-Verlag.

Kutner M, Nachtsheim C, Neter J, and Li W 2005. Applied Linear Statistical Models. Fifth ed. McGraw-Hill Irwin, New York.

Mills T 2009. Forecasting obesity trends in England. J. R. Statist. Soc. A 172, Part 1: 107-117.
