# Supplementary material for: Projection scenarios of body mass index (2013–2030) for Public Health Planning in Quebec
Source: BMC Public Health. 2014 Sep 25;14:996. doi: 10.1186/1471-2458-14-996 (PMC4196088; doi:10.1186/1471-2458-14-996)
Supplement: Supplementary file 2 — Additional file 2: Prediction Intervals, Goodness of Fit and Sensitivity Analyses. (DOC 442 KB) [file 12889_2014_7135_MOESM2_ESM.doc]

Additional file **2: Prediction Intervals, Goodness of Fit and Sensitivity Analyses**

1. Estimation of Prediction Intervals

The prediction variance of a projected BMI prevalence at time
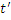
 is the sum of variance in estimated mean response and future measurement error (Kutner M et al. 2005):
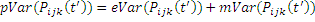
, where the prevalence specific to BMI category
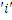
, age group
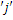
, sex
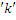
 and measured from survey timepoint
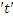
 is considered.

The covariance matrix of the estimated mean value of
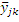
 at future time
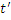
 (represented by
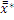
) is calculated using standard GLS (generalized least squares) theory:
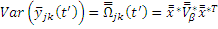
, where
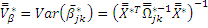
 is the covariance matrix of the estimated regression coefficients. The covariance matrix of the corresponding backtransformed prevalence composition at time
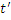
, is then estimated using the delta method:
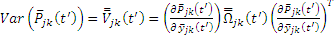
. The variance in estimated mean response,
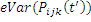
, is obtained from the diagonal elements of
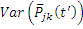
.


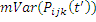
 is estimated by the variance structure of the 2012 CCHS, under the approximation that the survey design and sampling size of a future survey at time
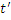
 will be similar:
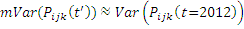
.

The prediction intervals for the projected prevalence of BMI category
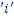
, age category
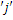
, sex
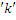
 at future time
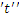
 are then estimated using a Normal approximation, by:
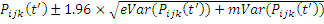
.

Prediction intervals represent estimates of the statistical uncertainty caused by variability in the measured data and the model estimation process. Prediction intervals in projection analyses are always underestimates of the actual error since they do not account for the structural error component, and thus they should not be interpreted as reliable bounds on estimated future values (Hakulinen et al. 1986; Moller et al. 2005). Prediction intervals are useful however for indicating the statistical stability of the projection model as well as providing a lower bound on the actual error. Prediction intervals for age aggregated BMI prevalence projections are shown in Figure A2 below.

**Figure A2 Projections (2013 to 2030) of age-aggregated prevalence by BMI category, for men and women. The linear scenario is indicated by the black line (―), the deceleration scenario is indicated by the gray line (―), and the historical BMI time series data are indicated by the open circles (○). The dotted black (…) and gray (…) lines indicate prediction intervals for the linear and deceleration scenarios. The blue (―) and red (―) lines indicate the sensitivity analysis results for the linear and deceleration scenarios.**


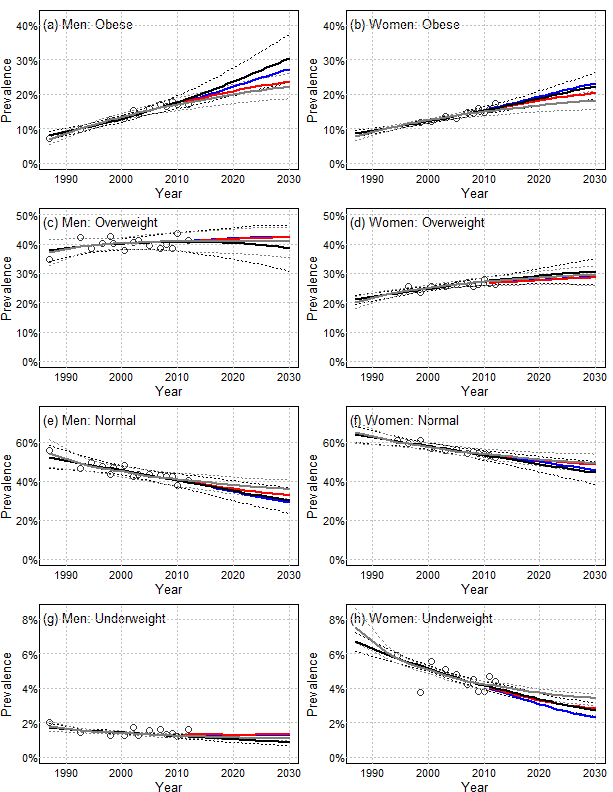


2. Goodness of Fit

Visual Assessment

Both linear and log models appeared to fit the age-aggregated (Figure 1 of the main text) and age-category specific (Appendix 4) measured prevalence trends well. The fitted models followed trends that were clearly apparent in the measured data, and residuals did not indicate the presence of any systematic bias.

Quantitative Assessment

Buse 1973 (Buse A 1973) derived the
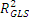
 statistic to assess goodness of fit for a GLS model that accounts for the weighting matrix in computing the residual sums of squares and the mean response:


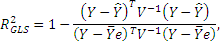


where
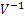
 is the weighting matrix,
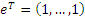
, and
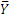
 is the weighted mean of the response variable:
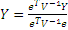
. This measure is analogous to the OLS (ordinary least squares)
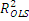
, and measures the proportion of the generalized sums of squares attributable to the regression model.
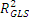
 ranges from 0 to 1 and reduces to the
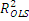
 when the weighting matrix is identity.
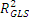
 was computed for each of the eight age by sex regression analyses, and for both linear and deceleration scenarios, as shown in Table A2.1 below. All values were found to be
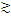
 0.96 indicating goodness of fit.

**Table A2.1**


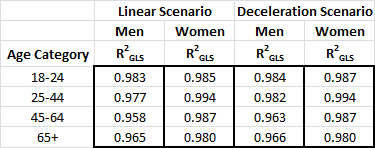


However as each regression analysis comprises fitting to all 3 transformed BMI categories simultaneously, this can lead to large and uninformative values of
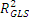
 as the between BMI category variation can mask within BMI category variation. Thus
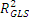
 was further applied to assess goodness of fit within the obese BMI category only, using subsets of the measured and fitted backtransformed data and covariance matrices. Results are shown in Table A2.2 below. It can be seen that
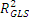

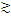
 0.44 for fitted obesity trends in all age and sex categories, except for 18-24 year old women. As can be seen in Appendix 3, Figure A3.1 however, the low values of
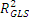
 for this stratum are due to the ‘flatness’ of the obesity trend and consequent low percentage of variability explained by the fitted trend, rather than poor goodness of fit.

**Table A2.2**


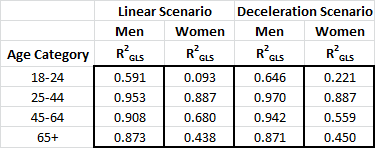


3. Sensitivity Analyses

Sensitivity analyses were performed to assess the robustness and validity of the projection results. These analyses comprised estimations of projected age-aggregated prevalence trends that used only the seven CCHS general health surveys from 2000-2001 to 2012. The purpose of the analyses was to assess primarily if projected trends might change if only the more recent historical data were used, and thus if the older historical data might be exerting undue influence on the projections. The analysis was further used to examine if additional stabilisation or ‘levelling-off’ in recent obesity prevalence trends might be present, as well as if the analysis of a more homogeneous time series might yield different results.

Results of the sensitivity analyses are shown superimposed on projection results, in Figure A2. It can be concluded that the projection results are robust to the use of more recent historical data. Projected trends using the data subset follow the full dataset projections. In particular, the projected obesity prevalence spanned by the linear and deceleration scenarios using the data subset lie within the span projected by the full dataset, for both men and women.

References

Buse A 1973. Goodness of Fit in Generalized Least Squares Estimation. The American Statistician 27: 106-108.

Hakulinen,T., Teppo,L., and Saxen,E. 1986. Do the predictions for cancer incidence come true? Experience from Finland. Cancer. 57: 2454-2458.

Kutner M, Nachtsheim C, Neter J, and Li W 2005. Applied Linear Statistical Models. Fifth ed. McGraw-Hill Irwin, New York.

Moller,B., Weedon-Fekjaer,H., and Haldorsen,T. 2005. Empirical evaluation of prediction intervals for cancer incidence. BMC. Med Res Methodol. 5: 21.
