# Supplementary material for: Projection scenarios of body mass index (2013–2030) for Public Health Planning in Quebec
Source: BMC Public Health. 2014 Sep 25;14:996. doi: 10.1186/1471-2458-14-996 (PMC4196088; doi:10.1186/1471-2458-14-996)

Additional file **3: Projection of type 2 diabetes: (1) separation of epidemiologic and demographic components, and (2) survey estimates of BMI, age and sex-specific prevalence of type 2 diabetes**

1. Separation of epidemiologic and demographic components

As described in the Methods (Projected impact of BMI on chronic disease prevalence) section of the manuscript, the projected BMI, age and sex specific numbers of cases of type 2 diabetes (
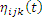
) is equal to the product of the type 2 diabetes prevalence in 2011-2012 (
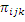
) and projected numbers of individuals (
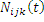
) corresponding to the same category.
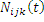
 itself is the product of the projected BMI prevalence (
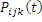
) and population (
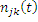
). Thus,


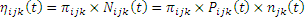


Total projected numbers of cases (
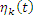
) and prevalence (
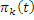
) by sex is obtained by aggregation over BMI and age categories as was described in the main manuscript (Methods, Projected impact of BMI on chronic disease prevalence). The equations for
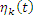
 and
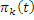
 are shown below, showing the respective dependence of these quantities on BMI prevalence (termed the epidemiologic component) and population projections (the demographic component):


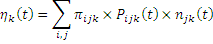


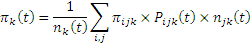


Estimation of the demographic contribution to type 2 diabetes numbers (
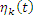
) and prevalence (
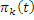
) is done by holding the age and sex specific BMI prevalences at a fixed reference level
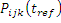
 and allowing only the population to evolve. Thus:


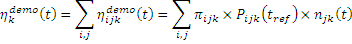


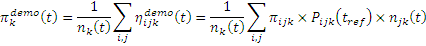


The epidemiologic contribution (due to BMI related change) is estimated by subtracting the demographic effect from the overall trends for both numbers and prevalence. Thus


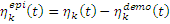


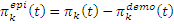


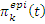
 can be interpreted as the component of change in prevalence amenable to intervention on population BMI, and
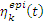
 as the estimated number of avoidable cases of type 2 diabetes.

2. 2011-2012 cross-sectional survey estimates of BMI, age and sex-specific prevalence of type 2 diabetes

In the current projections, the BMI, age and sex specific prevalence of type 2 diabetes (
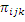
) is estimated from the 2011-2012 Canadian Community Health Survey (CCHS), and is assumed to remain constant over the projected time horizon.

Figures A3a and A3b show plots of the values of
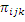
 for men and women respectively. BMI categories are indicated on the x-axis and the age categories are represented by separate curves. The marked increase in type 2 diabetes prevalence with BMI can be seen by the upward trends in the curves for both men and women. There is also a substantial increase in prevalence with age, as shown by the separation of the different curves in both plots. Thus it is expected that increasing BMI and population aging will likely combine to drive projected future increases in type 2 diabetes prevalence.

Figure A3, Type 2 diabetes prevalence vs. BMI plotted by age, for (a) Men and (b) Women.


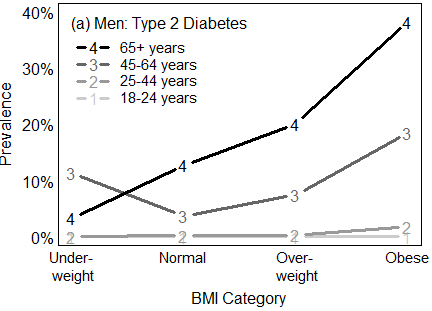

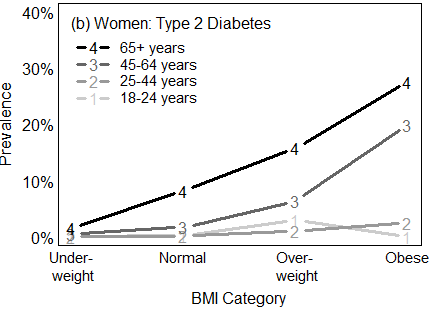

Supplement: Supplementary file 3 — Additional file 3: Projection of type 2 diabetes: (1) separation of epidemiologic and demographic components, and (2) survey estimates of BMI, age and sex-specific prevalence of type 2 diabetes. (DOC 238 KB) [file 12889_2014_7135_MOESM3_ESM.doc]
