# Supplementary material for: Projection scenarios of body mass index (2013–2030) for Public Health Planning in Quebec
Source: BMC Public Health. 2014 Sep 25;14:996. doi: 10.1186/1471-2458-14-996 (PMC4196088; doi:10.1186/1471-2458-14-996)

Additional file **4: Age-specific BMI prevalence projections**

**Figure A4.1, Projections of obesity prevalence by age category and sex for men and women. The linear scenario is indicated by the black line (―), the deceleration scenario is indicated by the gray line (―), and the historical BMI time series data are indicated by the open circles (○). The dotted black (…) and gray (…) lines indicate prediction intervals for the linear and deceleration scenarios.**


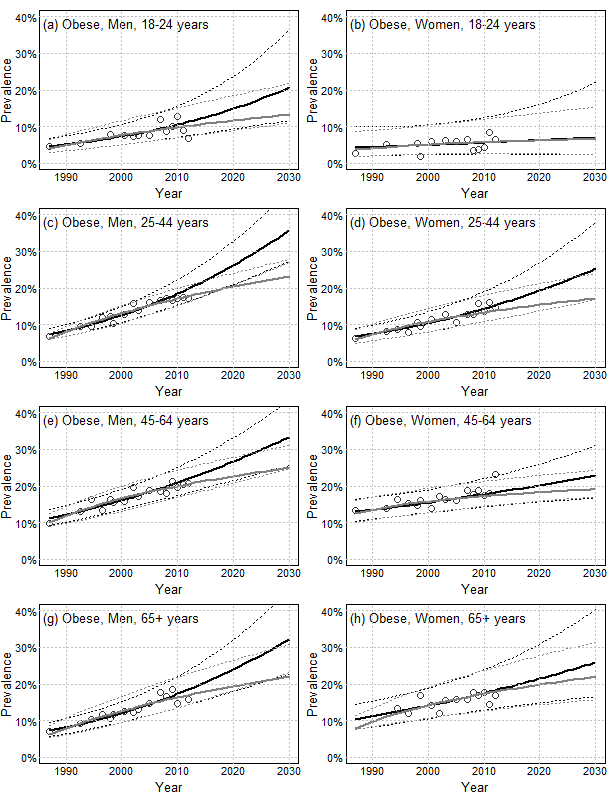


**Figure A4.2, Projections of overweight prevalence by age category and sex for men and women. The linear scenario is indicated by the black line (―), the deceleration scenario is indicated by the gray line (―), and the historical BMI time series data are indicated by the open circles (○). The dotted black (…) and gray (…) lines indicate prediction intervals for the linear and deceleration scenarios.**


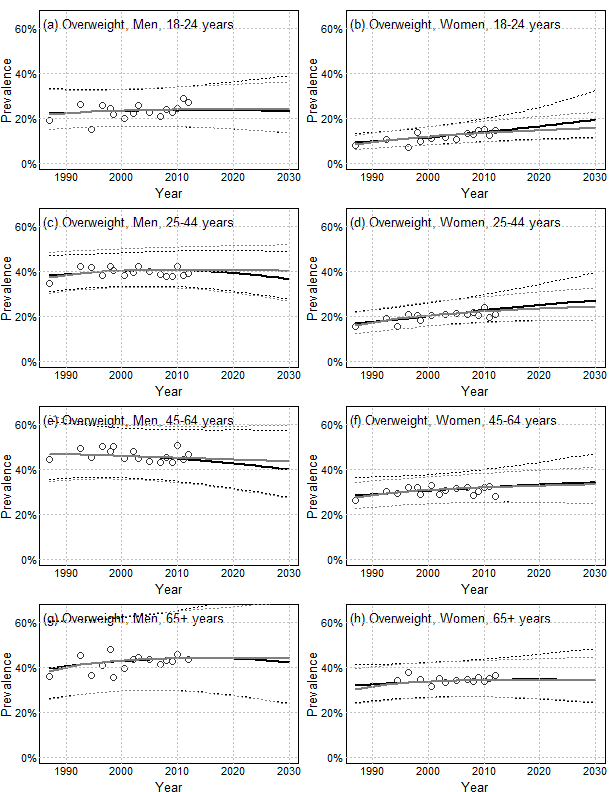


**Figure A4.3, Projections of normal weight prevalence by age category and sex for men and women. The linear scenario is indicated by the black line (―), the deceleration scenario is indicated by the gray line (―), and the historical BMI time series data are indicated by the open circles (○). The dotted black (…) and gray (…) lines indicate prediction intervals for the linear and deceleration scenarios.**


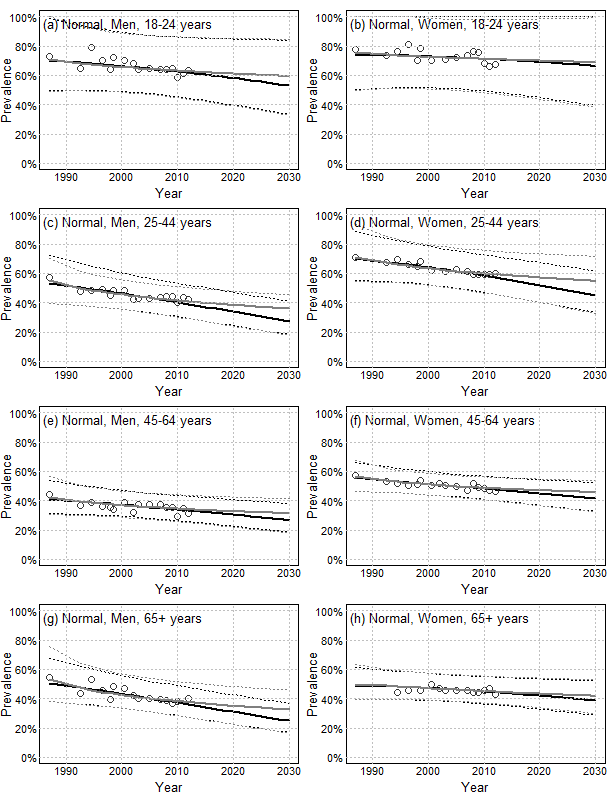


**Figure A4.4, Projections of underweight prevalence by age category and sex for men and women. The linear scenario is indicated by the black line (―), the deceleration scenario is indicated by the gray line (―), and the historical BMI time series data are indicated by the open circles (○). The dotted black (…) and gray (…) lines indicate prediction intervals for the linear and deceleration scenarios.**


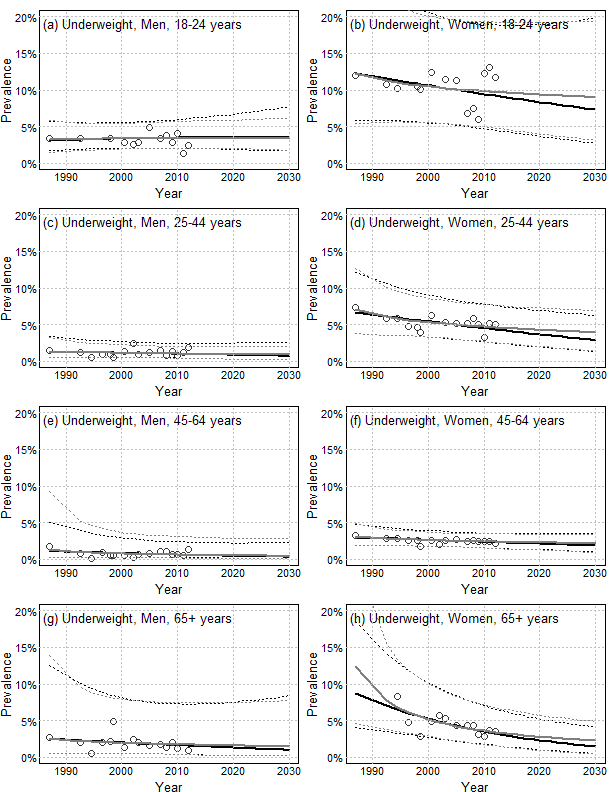

Supplement: Supplementary file 4 — Additional file 4: Age-specific BMI prevalence projections. (DOC 213 KB) [file 12889_2014_7135_MOESM4_ESM.doc]
